# Primary Mucinous Adenocarcinoma of the Testis

**DOI:** 10.1155/2012/685946

**Published:** 2012-04-09

**Authors:** Takeshi Azuma, Yukihide Matayoshi, Yasushi Nagase

**Affiliations:** Department of Urology, Tokyo Metropolitan Tama Medical Center, 2-8-29 Musashidai, Fuchu-shi, Tokyo 183-8524, Japan

## Abstract

Ovarian-type surface epithelial neoplasms of the testis and paratestis are uncommon, and the mucinous subtype is particularly rare. These tumors represent a counterpart to ovarian cancer. Malignant tumors have the potential for metastatic spread and are often fatal. The case of a 59-year-old man with testicular mucinous adenocarcinoma is presented. Computed tomography indicated involvement of the paraaortic and pelvic lymph nodes, so chemotherapy was initiated. To the best of our knowledge, this is the second paper regarding responsiveness to chemotherapies used in ovarian cancer.

## 1. Introduction

 Primary ovarian-type surface epithelial carcinomas of the testis and paratestis are very rare [1]. The mucinous subtype is particularly rare, with only 20 cases reported to date [2–6].

 We report a case of primary mucinous adenocarcinoma of the testis with retroperitoneal metastasis. We discuss the management of this uncommon testicular tumor based on the limited reports.

## 2. Case Report

**Figure 1:**
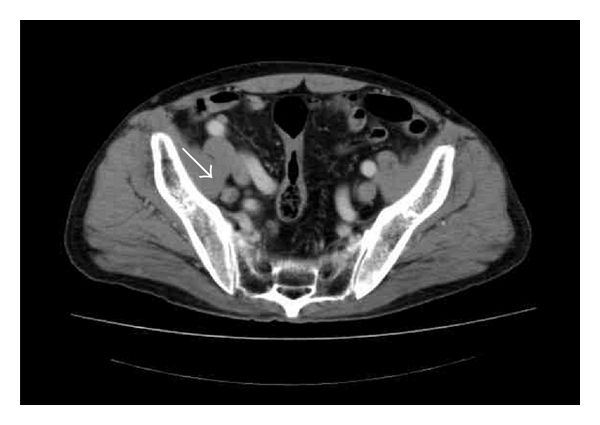
Computed tomography revealing metastases in pelvic lymph nodes.

A 59-year-old man with hyperlipidemia presented with a 2-month history of right testicular discomfort and painless swelling. Physical examination revealed a firm mass with an irregular surface in the right scrotum. No lymphadenopathy was palpable. Laboratory findings showed elevation of serum carbohydrate antigen 19-9 (CA19-9) to 17,200 U/mL (normal, <37 U/mL) and carcinoembryonic antigen (CEA) to 6.5 ng/mL (normal, <5 ng/mL), although levels were normal for serum germ cell tumor markers such as lactate dehydrogenase (LDH), *β*-human chorionic gonadotropin (*β*-hCG), and *α*-fetoprotein (AFP). Transscrotal ultrasonography showed a 6 × 4 × 2 cm mass with cyst in the right testis. Abdominal computed tomography (CT) revealed swelling of the paraaortic and pelvic lymph nodes to a diameter of 2.5 cm (Figure 1). Chest radiography showed no normal abnormalities. No gastrointestinal tumor was indicated on upper and lower gastrointestinal endoscopy.

**Figure 2:**
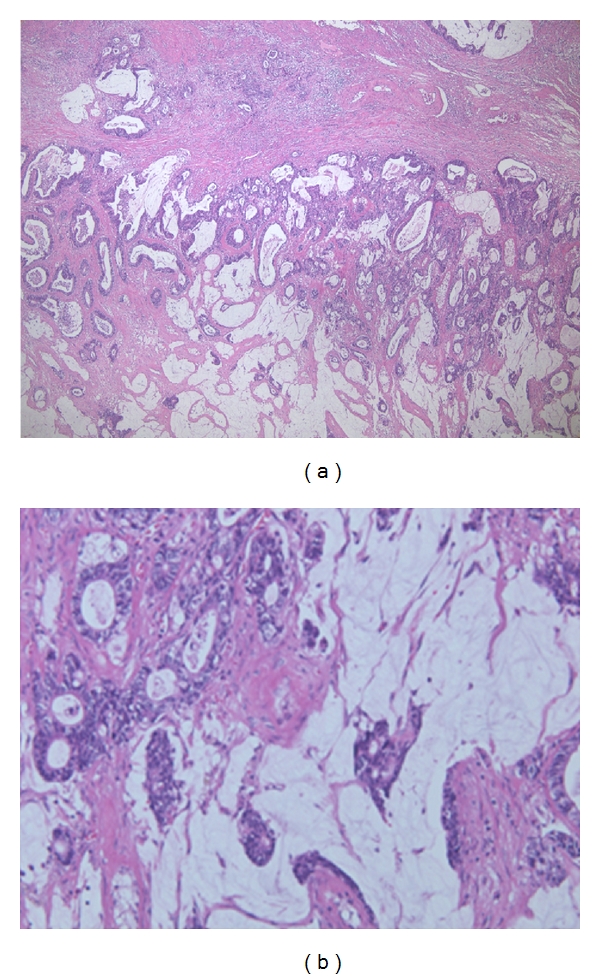
Microscopic findings (hematoxylin and eosin) showing mucinous adenocarcinoma. Single or multiple layers of columnar epithelium with mucin are visible. In some portions, papillary growth was also observed. (a) 40x; (b) 200x.

Right primary testicular tumor was diagnosed and the patient underwent right inguinal orchiectomy. Pathological examination demonstrated that the tumor consisted of solid tumor containing mucinous fluid. Microscopically, a single or several layers of columnar epithelium producing mucin showed papillary growth. The tumor epithelium exhibited stromal invasion (Figure 2). No teratomatous element, germ cell neoplasia, or ovarian-type stroma was evident.

After orchiectomy, serum CA19-9 fell to 6,860 U/mL. Adjuvant chemotherapy was initiated to treat paraaortic lymph node metastasis using paclitaxel (185 mg/m^2^) and carboplatin (AUC 6) administered every 3 weeks for a total of three cycles. After the first treatment, the level of CA19-9 halved but subsequently increased to 10,270 U/mL for the next two cycles. Multiple lung and liver metastases were identified on CT at this time. These findings suggested that the tumor had developed resistance to the first regimen. The patient decided against further treatment and died 10 months later.

## 3. Discussion

 Primary ovarian-type surface epithelial carcinomas of the testis and paratestis are rare [1]. Six subtypes have been defined: serous, mucinous, endometrioid, clear, transitional, and squamous. The most frequent of these is the serous subtype, while the mucinous subtype is very rare. To the best of our knowledge, only 20 cases (including the present case) have been reported to date [2–6]. The incidence of these tumors is higher among older age groups than that of common testicular tumors, with a median age at onset of 54 years [2–6]. Similar to ovarian tumors, the biological behavior ranges from benign to borderline to malignant with metastasis. Although the gross features resemble the well-known ovarian tumors, a few differences are worth noting. Testicular tumors are not generally as large as the ovarian ones, as the more conspicuous location results in patients seeking attention earlier. As a result, the chief complaint is commonly painless enlargement of the testis, sometimes with a hydrocele. Compared to an ovarian tumor, a testicular tumor is more often a unilocular cystic tumor.

The most important differential diagnosis is metastasis from the gastrointestinal tract for primary ovarian-type surface epithelial carcinomas of the testis. In cases with ovarian tumors, immunostaining for cytokeratin 7, MUC2, MUC5AC, and MUC6 has been reported to be useful in distinguishing this from metastatic ovarian tumors. Also, with intratesticular mucinous adenocarcinoma, some reports have suggested the utility of immunohistochemical staining [2]. However, solid evidence is lacking, because of unavailable material. The possibility of metastatic lesions from primary tumors of other primary sites should thus be excluded by appropriate clinical, endoscopic, and radiographic investigations.

The standard treatment for localized disease is radical orchiectomy. The significance of adjuvant chemotherapy remains controversial, as most reported cases have not shown metastases and have enjoyed a good prognosis. Our case displayed a metastasis at the time of first diagnosis of testicular tumor, and the tumor behaved in a positively malignant manner. We selected paclitaxel and carboplatin administered every 3 weeks as adjuvant chemotherapy, as used for first-line chemotherapy in ovarian cancer [7]. The tumor was initially sensitive to this regimen but became resistant after 3 cycles. The second-line agent for relapsed ovarian cancer is liposomal doxorubicin or gemcitabine. The patient desired palliative care and died 10 months after ending chemotherapy.

 In conclusion, our case showed two important points. The first point is that we should take into consideration that this type of tumor might have malignant potential, although most previous reports have shown the features of a favorable clinical outcome. Second, experience is limited with intratesticular mucinous adenocarcinoma. However, its behavior appears similar to that of its ovarian counterparts. Chemotherapy with paclitaxel and carboplatin might have potential efficacy, as previously reported.
